# On the Wrong Track: Alterations of Ciliary Transport in Inherited Retinal Dystrophies

**DOI:** 10.3389/fcell.2021.623734

**Published:** 2021-03-05

**Authors:** Laura Sánchez-Bellver, Vasileios Toulis, Gemma Marfany

**Affiliations:** ^1^Departament de Genètica, Microbiologia i Estadística, Universitat de Barcelona, Barcelona, Spain; ^2^Institute of Biomedicine (IBUB-IRSJD), Universitat de Barcelona, Barcelona, Spain; ^3^CIBERER, ISCIII, Universitat de Barcelona, Barcelona, Spain

**Keywords:** ciliopathy, inherited retinal dystrophies, intraflagellar transport, sensory cilium, photoreceptor, ciliary transport

## Abstract

Ciliopathies are a group of heterogeneous inherited disorders associated with dysfunction of the cilium, a ubiquitous microtubule-based organelle involved in a broad range of cellular functions. Most ciliopathies are syndromic, since several organs whose cells produce a cilium, such as the retina, cochlea or kidney, are affected by mutations in ciliary-related genes. In the retina, photoreceptor cells present a highly specialized neurosensory cilium, the outer segment, stacked with membranous disks where photoreception and phototransduction occurs. The daily renewal of the more distal disks is a unique characteristic of photoreceptor outer segments, resulting in an elevated protein demand. All components necessary for outer segment formation, maintenance and function have to be transported from the photoreceptor inner segment, where synthesis occurs, to the cilium. Therefore, efficient transport of selected proteins is critical for photoreceptor ciliogenesis and function, and any alteration in either cargo delivery to the cilium or intraciliary trafficking compromises photoreceptor survival and leads to retinal degeneration. To date, mutations in more than 100 ciliary genes have been associated with retinal dystrophies, accounting for almost 25% of these inherited rare diseases. Interestingly, not all mutations in ciliary genes that cause retinal degeneration are also involved in pleiotropic pathologies in other ciliated organs. Depending on the mutation, the same gene can cause syndromic or non-syndromic retinopathies, thus emphasizing the highly refined specialization of the photoreceptor neurosensory cilia, and raising the possibility of photoreceptor-specific molecular mechanisms underlying common ciliary functions such as ciliary transport. In this review, we will focus on ciliary transport in photoreceptor cells and discuss the molecular complexity underpinning retinal ciliopathies, with a special emphasis on ciliary genes that, when mutated, cause either syndromic or non-syndromic retinal ciliopathies.

## Introduction

The retina is a highly organized neurosensory tissue that covers the eye’s inner surface and is responsible for visual perception (den Hollander et al., 2010). In vertebrates, it consists of six major neural cell types that are arranged in laminar microcircuits necessary for the correct integration and processing of light signals. Photoreceptor cells, which occupy the outermost retinal layer, are light-sensitive neurons that capture photons and transduce light stimuli into an electrical signal, thereby triggering the phototransduction cascade. Phototransduction finishes when the electric signal is transmitted to the visual centers in the brain by the optic nerve, formed by the axons of ganglion cells (Wässle, 2004; Swaroop et al., 2010; Khanna, 2015). Two types of photoreceptor cells exist, rods and cones, the latter being outnumbered by approximately 20-fold in most mammalian species (Masland, 2001; Swaroop et al., 2010). Rods are responsible for dim light vision due to their high sensitivity to light, which enable them to capture even a single photon. In contrast, cones are less sensitive and are responsible for daytime vision and visual acuity. Cones also confer color perception because of the existence of several cone subtypes that selectively respond to photons in different wavelengths of the visible spectrum (Roorda and Williams, 1999; Deeb, 2006; Hoon et al., 2014).

Vertebrate photoreceptors are highly polarized and specialized neurons whose unique morphology includes a distinct compartment where photoreception and phototransduction occur: the outer segment. The outer segment is a specialized sensory cilium optimized for photon capture and efficient phototransduction. It contains membranous disks ordered in stacks filled with photoreceptive pigments (opsins), and is distinct between rods and cones in both structure and protein composition. However, the outer segment is devoid of protein synthesis and metabolism machinery, hence, these cellular functions occur in the inner segment and the components are transported to the distal outer segment by a microtubule ciliary gate, named connecting cilium (Khanna, 2015; Bujakowska et al., 2017). The tips of the outer segment are physically in contact with the retinal pigment epithelium, which constitutes the outermost blood-retina barrier and is involved in nourishment through the choroidal blood vessels, maintenance of photoreceptors, and mediation of the visual cycle (Strauss, 2005). Alterations, by either genetic or external factors in such a morphologically and functionally organized structure, can lead to photoreceptor dysfunction, eventually resulting in retinal degeneration and blindness (Wright et al., 2010).

## The Specialized Primary Cilium of Photoreceptor Cells

Cilia are microtubule-based organelles that project from the apical plasma membrane and play crucial roles in the development and function of adult organs in vertebrates. They are conventionally classified as motile or non-motile cilia. Motile cilia beat rhythmically to produce a driving force that either propel cells or move extracellular fluids across membrane surfaces (Ishikawa and Marshall, 2017; Viswanadha et al., 2017). Non-motile cilia, or primary cilia, act as cellular antennae that concentrate specific signaling receptors necessary for triggering signal transduction in response to external cues, such as morphogens, mechanical stimulation and light (Fliegauf et al., 2007; Goetz and Anderson, 2010). Photoreceptors possess highly specialized primary cilia that develop exceptional characteristics to carry out photoreception and phototransduction. Mutations in ciliary genes can compromise photoreceptor cilia structure, biogenesis and/or function, leading to retinal ciliopathies, which cover approximately one fourth of inherited retinal dystrophies (Chen et al., 2019; Wheway et al., 2019).

### Structure and Composition

**FIGURE 1:**
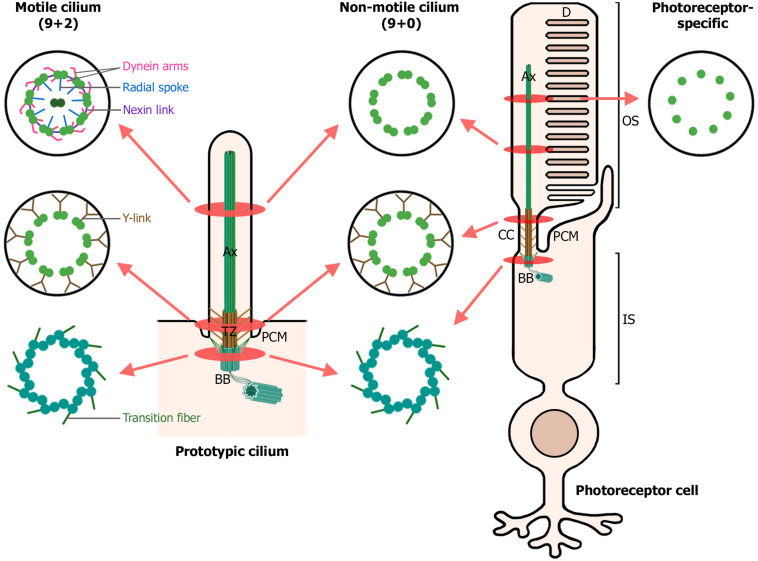
Structure of motile, non-motile, and photoreceptor cilia. The left diagram depicts a prototypic cilium with its main compartments: the axoneme (Ax), transition zone (TZ) with its Y-links, periciliary membrane (PCM) and the basal body (BB). The right diagram depicts a rod photoreceptor, which in addition, displays photoreceptor-specific cellular structures: the outer segment (OS), comprising the membranous disks (D) and the axoneme; the connecting cilium (CC), analogous to the transition zone in primary cilia; and the inner segment (IS), which contains the basal body and most cellular organelles. Cross-sections through the ciliary compartments of both motile and non-motile cilia are shown: the basal body triplet microtubules with their transition fibers, shared by motile cilia, non-motile cilia and photoreceptor primary cilia; the transition zone of motile and non-motile cilia and the analogous connecting cilium in photoreceptors; the axonemal microtubule doublets, decorated with dynein arms, radial spokes and nexin links in motile cilia; and finally, the axonemal microtubule singlets characteristic of photoreceptor primary cilia. (Figure created with BioRender.com).

Motile and primary cilia share a common structural composition and architecture, comprising three main distinct sub-compartments: the basal body, transition zone, and axoneme (Figure 1). Overall, their main difference resides in the axonemal microtubule organization. On one hand, motile cilia usually present 9 + 2 axonemes, with nine peripheral doublet microtubules and two central single microtubules. Furthermore, they possess additional associated components powering the ciliary movement, such as dynein arms, nexin links, and radial spokes (Viswanadha et al., 2017). In contrast, non-motile cilia axonemes typically lack the central pair of microtubules, the dynein arms, nexin links and radial spokes, being described as 9 + 0 axonemes (Figure 1). However, this classification is a simplification, as non-motile 9 + 2 and motile 9 + 0 cilia are also present in human cells (Fliegauf et al., 2007).

The primary cilium originates from the basal body, which is derived from the mother centriole and functions as the primary microtubule organizing center (MTOC), projecting also microtubules into the inner segment and cell body (Muresan et al., 1993; Petry and Vale, 2015). The basal body is composed of nine triplet microtubules, subdistal appendages and nine distal appendages, also known as transition fibers, which anchor the basal body to the periciliary membrane (Figure 1). The periciliary membrane corresponds to the border between the ciliary membrane and the cytoplasmatic membrane. The periciliary region surrounds the basal body and is a hot spot for endocytosis of ciliary membrane proteins. Together with transition fibers, the periciliary region serves as a docking station for proteins destined to the cilium (Reiter and Leroux, 2017; Seo and Datta, 2017). Furthermore, the basal body of primary cilia nucleates the ciliary rootlet. In photoreceptors, both the basal body and the ciliary rootlet anchor their exceptionally large cilium to the inner segment, providing long-term stability and structural integrity to this microtubule-based structure (Figure 1) (Yang et al., 2005; May-Simera et al., 2017). Ciliary rootlets are composed of rootletin fibers, encoded by the *CROCC* gene, which polymerize into thick filaments that are attached to the basal body by CEP250, mutations of which have been associated with Usher syndrome (USH) and retinitis pigmentosa (RP) (Yang et al., 2002, 2006; Khateb et al., 2014; De Castro-Miró et al., 2016).

The photoreceptor connecting cilium, equivalent to the transition zone in primary cilia, is localized to the junction between the basal body and the axoneme, linking the inner and outer segments of photoreceptor cells (Figure 1). It acts as a diffusion barrier, preventing abnormal mixing of ciliary and cytosolic components, and probably as a ciliary gate, regulating protein influx and efflux from the cilium. The connecting cilium possesses a different microtubule organization, and changes from triplets at the basal body to microtubule doublets (de Robertis, 1956; Cohen, 1965; Bachmann-Gagescu and Neuhauss, 2019), the latter being cross-linked to the surrounding plasma membrane by Y-link structures (Gilula and Satir, 1972). Interactome analysis in primary cilia have allowed to identify several protein complexes specific of the transition zone, which have been later described in photoreceptor connecting cilium. For instance, the Y-linker residing protein CEP290 (Rachel et al., 2015) associates into the NPHP module (RPGRIP1, RPGRIP1L, NPHP1, and NPHP4) and the MKS module (CC2D2A, B9D1, B9D2, MKS1, AHI1, TCTNs, and TMEMs). Mutations altering NPHP and MKS protein complexes cause syndromic ciliopathies (see section “Retinal ciliopathies and defects in ciliary transport”). The inversin compartment (INVS, NEK8, NPHP3, ANKS3, and ANKS6) localizes immediately distal from the transition zone and interacts through INVS with the MKS and NPHP complexes, establishing a link between the three of them (Braun and Hildebrandt, 2017; Garcia-Gonzalo and Reiter, 2017). Interestingly, mutations in components of the distinct protein modules NPHP, MKS or inversin result in dissimilar clinical phenotypes, which discloses the separated identities of each complex and the importance of their different roles in ciliary function.

The axoneme is the cytoskeleton backbone of the cilia. In photoreceptors, axonemal microtubules extend from the connecting cilium microtubule doublets to the outer segment, and become singlets near the tip in opposition to prototypic primary cilium axoneme, characterized by a doublet microtubule structure (Figure 1) (Cohen, 1965). Even though the axoneme is very stable, the distal (plus) ends of microtubules are constantly turned over and require a continuous delivery of tubulin to maintain axonemal length (Rosenbaum and Witman, 2002). The axoneme serves as railways for intraflagellar transport (IFT), which is responsible for the active bidirectional transport of many outer segment components along photoreceptor cilia, thus playing an essential role in cilium biogenesis, maintenance and function (Li et al., 2016).

Photoreceptor outer segments are packed with proteins involved in the phototransduction cascade, such as opsins (which belong to the family of G-protein coupled receptors, GPCRs), cGMP phosphodiesterases and cGMP-gated channels, but also with proteins that guarantee the maintenance of the particular architecture of the membranous disks, including PRHP2 and ROM-1 (Wensel et al., 2016). RP1, a photoreceptor-specific protein, associates with axonemal microtubules and is required for proper stacking of membranous disks. Mutations in *RP1* cause autosomal dominant RP (Liu et al., 2002, 2003, 2004). Surrounding the axoneme, there is the ciliary membrane, which is continuous with the plasma membrane. Protein and lipid content of the photoreceptor ciliary membrane are different from those of the surrounding plasma membrane in order to confer the ideal characteristics for an efficient signaling (Garcia et al., 2018).

**FIGURE 2:**
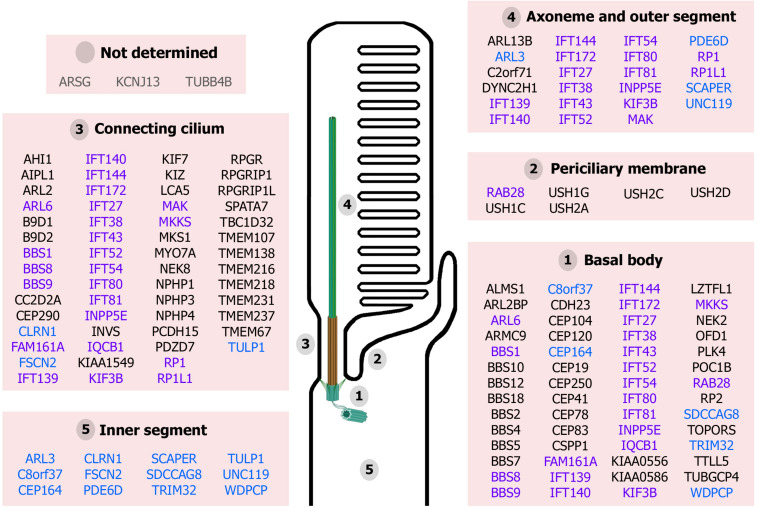
Proteins associated with retinal ciliopathies and their reported localization at the photoreceptor structures. Ciliary proteins can localize to unique ciliary subcompartments (names in black), several ciliary subcompartments (highlighted in purple) or other cellular compartments in addition to the cilium (in blue). The localization of ARSG, KCNJ13, and TUBB4B has not been determined yet (in gray). (Figure created with BioRender.com).

Finally, the cilium is a highly organized structure comprising distinct functional modules required for its proper function. Mutations in ciliary genes can result in protein disfunction and mislocalization. Thus, considering the extremely complex cilium of photoreceptor cells, mutations in these genes can potentially compromise photoreceptor cilium structure, biogenesis, function and maintenance, leading to retinal ciliopathies (Figure 2).

### Ciliogenesis

In undifferentiated progenitor photoreceptor cells, the first step of outer segment formation is ciliogenesis, followed by disk morphogenesis. In contrast to other organelles, cilia biogenesis and cell division are mutually exclusive in vertebrates. This is due to the intimate connection between cilia and centrosomes, which need to be released from the plasma membrane to operate during cell division, entailing ciliary resorption. Therefore, in replicative cells, ciliogenesis is highly coordinated with the cell cycle and requires a finely tuned regulation (Quarmby and Parker, 2005; Ishikawa and Marshall, 2011). However, many cells, including photoreceptors, do not undergo this cilium assembly disassembly cycle but grow instead a cilium after escaping cell cycle and entering into quiescence or differentiation (Toulis and Marfany, 2020).

It is well established that in primary cilia, ciliogenesis starts with the maturation of a mother centriole into a basal body due to the recruitment of the five distal appendage proteins. Therefore, even though the basal body consists of the mother and daughter centrioles, only the mother centriole, displaying distal and subdistal appendages, is further remodeled to enable cilium formation (Pearring et al., 2013). Rab8-carrying vesicles, derived from the Golgi and the recycling endosome, attach to the distal appendages of the mother centriole. SNAP29, a SNARE regulator of membrane fusion, promotes vesicular assembly that induces the conversion of small ciliary vesicles into a larger vesicle on the distal end of the mother centriole (Lu et al., 2015). The basal body migrates toward the apical end of the inner segment, where it docks to the cell membrane via the ciliary vesicle (Sánchez and Dynlacht, 2016). Then, microtubule doublets emanate from the basal body and form the proximal axoneme where the transition zone complexes tether, resulting in the assembly of the connecting cilium. The growth of the axoneme causes the invagination of the ciliary vesicle and its fusion with the plasma membrane, terminating in the emergence of the cilium, enclosed by the ciliary membrane, to the extracellular space (May-Simera et al., 2017). Tubulin subunits are actively transported via anterograde IFT into the ciliary compartment, leading to further extension of the axoneme by its distal end. Thus, complete extension of the axoneme relies on the IFT machinery to traffic ciliary components to the cilium tip forth and back, as well as the availability of soluble tubulin and axonemal precursors.

### Disk Morphogenesis and Renewal

The photoreceptor outer segment displays ordered stacks of tightly packed membranous disks. So far, two hypotheses have been postulated to explain outer segment disk morphogenesis in rod photoreceptors. The first proposed model is the evagination model. It postulates that disks are generated from outgrowths of the ciliary membrane at the outer segment base, which then extend away from the axoneme and appear as open disks, exposed to the extracellular milieu. Next, the basal membrane of an older evagination and the apical membrane of the adjacent younger evagination fuse at the disk hairpin-like rims, at the opposite side of the axoneme, resulting in the formation of a new enclosed disk (Steinberg et al., 1980). The alternative model, the vesicular targeting model, proposes that disk morphogenesis is caused by the repeated fusion of endocytic vesicles at the outer segment base and the following flattening of the primitive disk sacs to generate the mature disks. Vesicles may be derived either from the plasma membrane of the outer segment or from transport vesicles shipped from the inner segment through the connecting cilium (Chuang et al., 2007). Recent publications have provided strong evidence supporting the evagination model for the formation of vertebrate photoreceptors (Farjo et al., 2006; Burgoyne et al., 2015; Goldberg et al., 2016).

Efforts have been made to identify key molecules involved in disk morphogenesis, and defects in this process lead to non-syndromic retinal degeneration. In vertebrate photoreceptors, filamentous actin (F-actin) is mostly present in the inner segment as a submembrane network whereas outer segment seems to be deficient in an actin cytoskeleton except for the basal part of the outer segment (Chaitin et al., 1984; Lewis et al., 1995). There, F-actin and actin-associated proteins appear to be required for initiation of the evagination of the ciliary membrane but not for the continued growth of new evaginations (Williams et al., 1988; Hale et al., 1996; Corral-Serrano et al., 2020). Peripherin-2 (PRPH2/RDS) is a transmembrane glycoprotein and a key element for outer segment morphogenesis. PRPH2/RDS strongly interacts with ROM-1, which is also found in disk rims (Bascom et al., 1992; Moritz and Molday, 1996). PRPH2/RDS homomeric complexes are required for membrane geometry of disk rims, whereas PRPH2/ROM-1 complexes are involved in the proper stabilization and structuration of the outer segment. ROM-1 is not essential for outer segment morphogenesis but it is needed for photoreceptor viability (Stuck et al., 2017). Prominin-1 (PROM1) is a transmembrane protein that probably participates in disk morphogenesis regulation, maintaining the open structure of photoreceptor disks (Han et al., 2012). Finally, rhodopsin is the most abundant protein in rod outer segments, comprising almost 50% of the outer segment membrane mass. Consequently, in addition to being the photon receptor and activating the G protein cascade of phototransduction, rhodopsin is considered to have a structural role, serving as an indispensable element for the architecture of the rod outer segment membrane (Wensel et al., 2016).

The process of membranous disk morphogenesis remains after outer segment development and maturation due to the fact that it is continuously regenerated to minimize the accumulation of damaged molecular components due to photooxidative stress. Thus, outer segment length is maintained by the balance between basal disk formation and distal disk shedding. This ongoing renewal is a unique characteristic of photoreceptors and, 8–10% of the more distal disks are phagocytosed by the retinal pigment epithelium, and renewed on a daily basis (Young, 1967).

Clearly, outer segment renewal requires a high rate of delivery of freshly synthesized proteins to nascent disks, hence the critical role of ciliary transport not only for photoreceptor development but also for photoreceptor survival.

## Ciliary Transport in Photoreceptors

Several studies have been conducted to unravel protein sorting and transport to the primary cilium. On the other hand, the photoreceptor outer segment has shown to be a prolific model for the investigation of protein targeting to cilia due to its highly elevated rate of continuous renewal. Consequently, photoreceptor outer segment is the region with the highest demand for protein, yet all protein synthesis occurs in the inner segment. Thus, all components required for outer segment formation, maintenance and function must be transported from the IS to the outer segment through the connecting cilium. This transport demands stringently regulated mechanisms to enable efficient trafficking of selected proteins for the establishment and maintenance of photoreceptor identity. Any alteration in either cargo delivery to the cilium or ciliary transport compromises photoreceptor function and survival leading to retinal degeneration.

In photoreceptors, delivery of proteins to the outer segment can be conducted by different mechanisms. Ciliary trafficking powered by the IFT machinery is instrumental for opsin delivery to the outer segment, but chaperone-assisted transport and diffusion also play a relevant role in protein transport and delivery to the photoreceptor outer segment.

### Transport of Membrane Proteins With Cilia-Targeting Motifs: The Paradigm of Rhodopsin

Rhodopsin transport to the cilium has been extensively studied as it is the main cilia-targeted cargo protein in rods, with approximately 1000 rhodopsin molecules being trafficked through the connecting cilium per second (Besharse, 1986). Most of what is currently known of the transport and targeting of proteins to the photoreceptor outer segment stems from rhodopsin studies.

Similar to other membrane proteins, rhodopsin is transported by a conventional secretory pathway regulated by small GTPases, which function as molecular switches. Rhodopsin is synthesized at the rough endoplasmic reticulum and transported to the Golgi apparatus, where it is glycosylated. Then, rhodopsin exits the Golgi complex and reaches the *trans*-Golgi network (TGN), where it is specifically targeted and trafficked to the cilium (Deretic, 1998). In contrast to membrane transport between the endoplasmic reticulum and the Golgi mediated by the COPI and COPII coat proteins, rhodopsin transport from the TGN to the cilia is mediated by a different system (Lee et al., 2004).

Rhodopsin possesses a highly conserved VxPx motif in its C-terminus that is sufficient for its targeting to the OS (Chuang and Sung, 1998; Tam et al., 2000). This motif is also present in other ciliary proteins, such as polycystins 1 and 2, and the nucleotide-gated olfactory channel CNGB1b subunit (Geng et al., 2006; Jenkins et al., 2006; Ward et al., 2011). Rhodopsin contains an additional ciliary targeting signal in the cytoplasmic helix H8, the FR motif, which is also found in several other GPCRs (Corbit et al., 2005; May-Simera et al., 2017).

**FIGURE 3:**
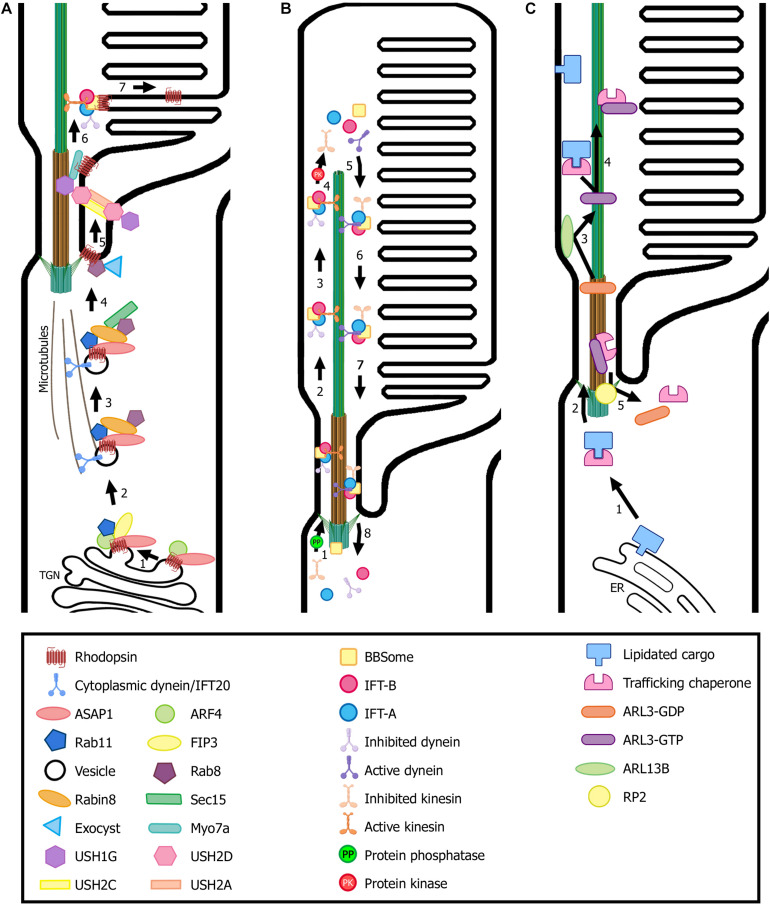
Transport of ciliary proteins in photoreceptor cells. **(A)** Rhodopsin is transported from the *trans*-Golgi network (TGN) to the rod outer segment by a conventional secretory pathway regulated by small GTPases. Many ciliary protein complexes are involved in this process, performing different roles in trafficking that enable rhodopsin docking and transport through the connecting cilium to the ciliary membrane. Note the location of the USH protein complex formed by USH1G, USH2D, USH2A, and USH2C subunits. **(B)** The proposed model for the cycle of the IFT complexes in photoreceptors is based on flagella and primary cilium as well as cryo-EM structural models (Liang et al., 2014; Roberts, 2018; Toropova et al., 2019). IFT-B trains (responsible for anterograde trafficking) are assembled at the base of the cilium and move along the outer segment axoneme to the tip, where they are disassembled. Then, IFT-A trains are assembled at the tip and enable retrograde trafficking until they reach the base of the cilium, where they are disassembled to begin another trafficking cycle. Numbers in each panel indicate the sequence of transport steps. In all images, only selected proteins are represented, for the sake of clarity. **(C)** Transport and delivery of lipidated proteins from the endoplasmic reticulum (ER) to the outer segment is assisted by trafficking chaperones (PDE6D and UNC119b) and regulated by small GTPases (e.g., ARL3). (Figure created with BioRender.com).

At the TGN and after maturation, rhodopsin is incorporated into rhodopsin transport carriers (RTCs) and a ciliary targeting module is formed. First, a member of the Arf family of G-proteins that regulate membrane transport, Arf4-GTP, binds the VxPx motif, and the Arf GTPase activating protein (GAP) ASAP1 recognizes the FR motif. Next, FIP3 and Rab11-GTP are recruited to the complex by ASAP1, forming the ciliary targeting module. FIP3 is a dual Arf/Rab11 effector that may function as a coordinator of Arf4 and Rab11 activities while Rab11 is a small GTPase. ASAP1 induces membrane deformation through its BAR domain and, at the same time, mediates GTP hydrolysis of Arf4, resulting in the release of Arf4-GDP from the complex (Figure 3A) (Mazelova et al., 2009a; Deretic and Wang, 2012).

Once Arf4 exits the ciliary targeting module, the rhodopsin C-terminal cytoplasmic tail is free to bind the dynein light chain Tctex-1. Then, cytoplasmic dynein carries RTCs from the Golgi to the base of the cilium along the microtubules, which nucleate from the basal body, toward their minus ends (Tai et al., 1999).

Along the vesicle’s route toward the basal body, ASAP1 recruits the small GTPase Rab8-GDP and its guanine nucleotide exchange factor (GEF), Rabin8, to the complex (Figure 3A). Rabin8 then promotes nucleotide exchange on Rab8-GDP, thus becoming Rab8-GTP (Deretic and Wang, 2012; Pearring et al., 2013). Interestingly, in addition to Rabin8, photoreceptors present a supplementary Rab8 GEF, Retinitis Pigmentosa GTPase Regulator (RPGR), located at the connecting cilium. RPGR facilitates Rab8 GDP/GTP exchange at a slightly slower rate than that of Rabin8, and mutations that disrupt its GEF activity cause X-linked RP. The existence of two Rab8 GEFs implies that the OS may need additional levels of Rab8 control to maintain its elevated level of vesicular ciliary traffic, as activated Rab8 plays a critical role in docking and fusion of the RTCs at the periciliary membrane (Murga-Zamalloa et al., 2010). Activated Rab8 binds to the exocyst complex subunit SEC15, which then interacts with SEC10. SEC10 is associated with the rest of the exocyst complex. This results in bringing RTCs to the base of the cilium. The exocyst complex is placed there by the small GTPase Cdc42, and tethers to the periciliary membrane (Lobo et al., 2017; Malicki and Johnson, 2017). Vesicle fusion with the periciliary membrane is mediated by SNARE protein complexes, specifically Syntaxin 3 and SNAP25, by bridging the opposing membranes and bringing them into proximity (Mazelova et al., 2009b).

Other connecting cilium proteins have been observed to modulate Rab8 ciliary localization, such as CC2D2A and RPGRIP1 (Bachmann-Gagescu et al., 2011; Raghupathy et al., 2017). Most probably, CC2D2A provides a specific docking point for ciliary-targeting vesicles at the base of the cilium by organizing the vesicle fusion machinery at the periciliary membrane. First, Rab8, which is coating the RTCs, binds to MICAL3, which in turn associates with the centrosomal protein NINL. NINL also interacts with the cytoplasmic dynein complex, linking the carrier vesicles and the motor that is responsible for the movement along the microtubules. Then, NINL binds CC2D2A at the base of the connecting cilium, conferring the specificity of the docking station. Concurrently, CC2D2A localization to the connecting cilium is needed for proper positioning of SNAP25 to the periciliary membrane. Finally, MICAL3 redox activity induces the remodeling of the complex and results in vesicle fusion and cargo release into the periciliary membrane (Grigoriev et al., 2011; Bachmann-Gagescu et al., 2015b; Ojeda Naharros et al., 2017).

Furthermore, it has been proposed that IFT20 may indirectly bind Rab8 or interact with Rabin8, thus participating in the transport and delivery of ciliary membrane proteins, including rhodopsin, from the Golgi apparatus to the ciliary base. There, IFT20 and the protein cargo would engage with the rest of the IFT complex in order to transport the targeted proteins through the connecting cilium and into the photoreceptor outer segment via ciliary trafficking, which will be further explained below in section “IFT- and BBSome-Mediated Trafficking” (Sedmak and Wolfrum, 2010; Crouse et al., 2014; Wang and Deretic, 2014).

Rhodopsin molecules are further transported through the connecting cilium, within the ciliary membrane, to the site of disk morphogenesis. This transport may probably be conducted by the motor proteins kinesin-II, a microtubule-dependent anterograde IFT subunit, and myosin VIIa, an actin-dependent motor molecule (Figure 3A) (Liu et al., 1999; Wolfrum and Schmitt, 2000; Williams, 2002; Chadha et al., 2019). Recently, USH syndrome protein network at the periciliary region has been linked to anterograde IFT by direct interaction between USH1G (SANS) and the IFT-B complex subunits, further elucidating the role of USH protein complex in ciliary transport. The periciliary transmembrane proteins USH2A and USH2C (the latter also known as GPR98, VLGR1, or ADGRV1) bind to USH2D by their intracellular domains, and USH2D, in turn, interacts with USH1G in the cytoplasm of the inner segment (Figure 3A) (Maerker et al., 2008). USH1G had been previously associated with rhodopsin cargo vesicles by its interaction with cytoplasmic dynein (Papal et al., 2013) and a recent study showed that the USH protein complex would transfer cargos from the cytoplasmic dynein transport module to the ciliary transport machinery propelled by myosin VIIa and/or kinesin-II motors (Sorusch et al., 2019).

Additionally, an alternative or complementary molecular model was described for ciliary transport of membrane cargos, such as rhodopsin, through the connecting cilium. In this model, ciliary transport is mediated by a non-vesicular ternary import complex comprised by transportin1 (TNPO1), Rab8-GDP and the ciliary membrane cargo, localized to the periciliary membrane. First, Rab8-GDP binds the ciliary targeting signal of the targeted protein and this further recruits TNPO1 to the complex. Next, the ternary import complex translocates across the membrane diffusion barrier that represents the connecting cilium, this process being facilitated by TNPO1. After translocation, cilium-localized GEFs exchange GDP for GTP on Rab8, resulting in the disassembly of the ternary complex and the release of the cargo to the ciliary membrane (Madugula and Lu, 2016).

Therefore, it is plausible to think that the interplay and cooperation between discrete and dynamic protein complexes, which accomplish slightly distinct roles in ciliary transport, may culminate in the docking and transport of selected proteins along photoreceptor cilia.

### Transport of Membrane Proteins Lacking Cilia-Targeting Motifs

It was hypothesized the VxPx motif was a general ciliary targeting signal for membrane proteins in photoreceptors. Nevertheless, it has only been described for the photoreceptor-specific retinol dehydrogenase (prRDH), apart from the opsins (Luo et al., 2004). Thus, alternative motifs and transport pathways seem to be responsible for ciliary protein cargo delivery at the outer segment.

Unlike the nucleotide-gated olfactory channel CNGB1b subunit, the visual CNGB1a subunit lacks a VxPx motif and its transport to the outer segment is directed by Ankyrin-G, a membrane adaptor that directly binds the C-terminus of CNGB1a (Jenkins et al., 2006; Kizhatil et al., 2009).

Several outer segment proteins without ciliary targeting sequences are co-transported in a complex with rhodopsin. This is the case of the progressive rod-cone degeneration protein (PRCD), whose function is unknown, and guanylate cyclase-1 (GC-1), a critical component of the phototransduction machinery (Pearring et al., 2015; Spencer et al., 2016). In bovine retinal extracts, co-immunoprecipitation assays detected IFT-cargo complexes containing rhodopsin, GC1 and the chaperone proteins MRJ and HSC70. Both chaperones interact with IFT88, loading the protein cargo into the IFT-B trains for transport through the connecting cilium, as well as delivery to the nascent disks of photoreceptors (Bhowmick et al., 2009).

The abundant outer segment protein PRPH2 is mainly transported via an unconventional secretory pathway that bypasses the Golgi apparatus and, although its C-terminus is important for ciliary targeting, no targeting motif has been characterized so far, and no proteins capable of recognizing PRPH2 C-terminus have been described (Tian et al., 2014).

### IFT- and BBSome-Mediated Trafficking

First described in *Chlamydomonas* flagella (Kozminski et al., 1993), intraflagellar transport (IFT) is a conserved transport mechanism in a diversity of motile and non-motile cilia in eukaryotic organisms, including photoreceptor cells. This process is defined as the bidirectional motor-dependent cargo transport along the axonemal microtubules from the base to the tip of the cilia, known as anterograde IFT, and back to the base, called retrograde IFT. It is critical for cilia development, maintenance and length-control, as it guarantees the assembly and the molecular turnover of ciliary components (Sedmak and Wolfrum, 2010; Khanna, 2015).

#### Composition of IFT Trains

Intraflagellar transport particles are organized into two large multimeric protein complexes: IFT-A, which is responsible for retrograde transport and is powered by kinesin-2 motors, and IFT-B, which is responsible for anterograde transport and is carried by dynein-2 (Cole, 2003). IFT trains are formed by more than 20 central components, arranged in subcomplexes, and the BBSome. Therefore, cargo molecules that are transported via IFT comprise the IFT machinery itself, axonemal building blocks – including tubulin and signaling molecules – and the BBSome (Prevo et al., 2017).

IFT-B complex is divided into the core (IFT-B1) subcomplex, comprising ten subunits (IFT22, 25, 27, 46, 52, 56, 70, 74, 81, and 88) and the peripheral (IFT-B2) subcomplex, composed of six subunits (IFT20, 38, 54, 57, 80, and 172). IFT52, IFT88, IFT38, and IFT57 interactions connect both subcomplexes (Taschner et al., 2016; Nakayama and Katoh, 2018). IFT-B motors are members of the kinesin-2 family and are found in heterotrimeric and homodimeric forms. The heterotrimeric kinesin-2 is composed by two motor subunits, KIF3A and either KIF3B or KIF3C, and an accessory non-motor subunit, KAP3. KIF3B and KIF3C bind KIF3A but not each other and, unlike in other tissues, they have partially redundant functions in photoreceptor cilia (Malicki and Besharse, 2012). The homodimeric kinesin-2 consists of KIF17 and it seems to be involved in distal microtubule singlet assembly in vertebrate photoreceptors as it accumulates at the distal tip of the outer segment (Bader et al., 2012).

IFT-A complex consists of the core subcomplex (IFT122, 140, and 144) that interacts with the IFT43–IFT121 dimer via IFT122. IFT139 and TULP3 are positioned on opposite sides of the IFT-A complex, IT139 interacting with the IFT43–IFT121 dimer and TULP3 interacting with the core subcomplex (Nakayama and Katoh, 2018). IFT-A is powered by dynein-2, which comprises three types of light chain shared with other dyneins (DYNLRB, DYNLL, and DYNLT) and five specific components: the motor-domain-containing heavy chain (DYNC2H1), a light chain (TCTEX1D2), a light intermediate chain (DYNC2LI1) and two intermediate chains (WDR34 and WDR60) (Toropova et al., 2019).

#### The BBSome

The BBSome is a stable complex that includes eight proteins (BBS1, 2, 4, 5, 7, 8, 9, and 18), encoded by causative genes of Bardet–Bield Syndrome (BBS), and the small GTPase ARL6/BBS3, which connects the complex with the ciliary membrane by its interaction with BBS1 (Jin et al., 2010; Prevo et al., 2017).

Bardet–Bield Syndrome proteins are essential for both rod and cone photoreceptor structure, function and survival (Dilan et al., 2018). The BBSome functions as an adaptor between cargo and the IFT complex, being specially required for retrograde protein ciliary trafficking and generally dispensable for protein transport into the photoreceptor outer segment (Datta et al., 2015; Liu and Lechtreck, 2018).

Furthermore, it acts as a ciliary gatekeeper in photoreceptors by preventing accumulation of non-ciliary protein in the outer segment, ensuring the compartmentalized protein distribution in photoreceptors (Hsu et al., 2017). The BBSome is negatively regulated by LZTFL1, which binds the complex to the cytoplasm and prevents its entry into the cilium, thereby regulating ciliary transport (Seo et al., 2011).

On the other hand, GPCRs and other unwanted ciliary proteins are tagged with ubiquitin chains, which are then recognized by the BBSome and removed from cilia, as has been recently reported in mammalian photoreceptors and *Chlamydomonas reinhardtii* (Shinde et al., 2020). Additionally, the BBSome has also been suggested to have a role in photoreceptor synaptogenesis and axonal targeting in neurons (Hsu et al., 2020).

#### The IFT Cycle

In broad terms, the IFT cycle can be split into four phases: first, cargo is assembled onto IFT trains near the basal body of photoreceptor cilia; next, anterograde transport to the tip of the cilia takes place; then, the IFT-B particle is disassembled, cargo is exchanged and the IFT-A train is assembled; finally, retrograde transport, back to the basal body occurs (Pearring et al., 2013).

The photoreceptor periciliary region, near the basal body, is rich in IFT proteins where they may cooperate with elements of the BBSome, to sort and deliver the ciliary membrane proteins. There, the IFT-B complex assembles and active kinesin-2 motor drives the anterograde train through the transition zone and along axonemal acetylated microtubules to the ciliary tip (Nachury et al., 2010; Sedmak and Wolfrum, 2010). The IFT-B subunit IFT74 is required for the assembling of IFT-A and IFT-B into IFT trains (Brown et al., 2015). As shown in *Chlamydomonas* flagella, dephosphorylation of the KIF3B subunit results in the incorporation of active kinesin-2 motor into anterograde IFT particles whereas KIF3B phosphorylation disrupts the interaction between kinesin-2 and IFT particles, leading to IFT train dissociation and cargo release (Figure 3B) (Liang et al., 2014).

Inhibited dynein-2 is transported to the ciliary tip as a passive cargo of IFT-B. According to cryo-EM structural studies, disassembly of the anterograde IFT train at the ciliary tip results in the destruction of the binding site for inhibited dynein-2, which uncovers an activating binding site for the IFT-A motor and enables its reconfiguration for retrograde transport. Then, IFT subunits are reassembled into retrograde trains, which carry the IFT-B complex with inactive kinesin-2 as a passive cargo, and move toward the base of the cilium, where they are disassembled to start another cycle of transport (Figure 3B) (Roberts, 2018; Toropova et al., 2019). Recent studies in BBSome-defective cells have shown that the BBSome is required for retrograde trafficking and/or ciliary exit of GPCRs, whereas defects in this complex do not impair anterograde transport (Nakayama and Katoh, 2018; Nozaki et al., 2018).

In the conventional IFT pathway, the IFT cargo is transported all along the axoneme and delivered at the ciliary tip. In photoreceptors, however, the cargo is delivered and incorporated into the growing disks, which means that the IFT machinery is only required for cargo transport from the basal body to the base of the outer segment (Figure 3A). Then, IFT trains move beyond the outer segment base to permit the switch from kinesin to dynein motors at the axoneme tip. The ensuing return of the IFT complex to the basal body via retrograde transport enable the recycling of the IFT components (Pearring et al., 2013).

### Delivery of Lipidated Proteins to the Outer Segment

Lipid modifications enable the association of many outer segment signaling proteins with disk membranes. Transport and delivery of lipidated cargo proteins to the photoreceptor outer segment is assisted by lipid-binding proteins that function as trafficking chaperones, and enable the diffusion of lipidated proteins through the photoreceptor cytoplasm by protecting the lipid fraction within a hydrophobic pocket (Pearring et al., 2013). In photoreceptors, two trafficking chaperones, PDE6D and UNC119b, have been identified. PDE6D (also known as PrBP/δ, originally thought to be the δ-subunit of the cGMP phosphodiesterase PDE) interacts with prenylated proteins such as PDE6, and farnesylated proteins like GRK1 (Zhang et al., 2007). UNC119b is an acyl-binding protein that interacts with the α-subunit of transducin, Gα_*t*_ (Zhang et al., 2011).

After synthesis, lipidated proteins remain transiently associated with the endoplasmic reticulum where they are post-translationally modified. There, the trafficking chaperones UNC119b and PDE6D bind their respective cargoes and exit the endoplasmic reticulum in a soluble, diffusible complex toward the cilium (Constantine et al., 2012; Hanke-Gogokhia et al., 2016). Next, ARL3-GTP, which has been previously activated by its GEF, ARL13B, in the outer segment, is recruited to the complex (Gotthardt et al., 2015). Consequently, lipidated cargo leaves the complex and associates with the ciliary membrane (Fansa et al., 2016; Hanke-Gogokhia et al., 2016). In fact, binding of both small GTPases ARL3 and ARL2 can release cargo from PDE6D, whereas only ARL3 can bind and release protein cargo from UNC119b (Ismail et al., 2011, 2012). RP2, which is localized to the basal body, interacts with the complex comprised by the trafficking chaperone and ARL3-GTP, and functions as a GAP, inducing ARL3 GTPase activity. GTP hydrolysis results in complex dissociation and the trafficking chaperones and ARL3-GDP exit the cilium to begin another transport cycle (Figure 3C) (Wright et al., 2011; Gotthardt et al., 2015).

### Light-Dependent Translocation of Signaling Proteins Into the Outer Segment

As mentioned, different mechanisms are used to transport proteins to the photoreceptor outer segment. Signaling proteins that depend on the light-dark cycle are translocated to the outer segment by mediated-diffusion. The base of the outer segment might not act as a selective barrier for soluble proteins according to studies with transgenic animals expressing GFP, in which soluble proteins quickly equilibrated between the outer segment and the inner segment by free diffusion through the connecting cilium (Calvert et al., 2010). Interestingly, further research revealed that this passive diffusion of soluble proteins is conditioned by steric volume exclusion. This phenomenon postulates that the three-dimensional conformation of a given molecule and the architecture of the aqueous space differently reduces the aqueous volume available to the protein and, consequently, regulates its entrance to distinct cellular compartments (Najafi and Calvert, 2012; Najafi et al., 2012).

Due to the cytoplasmic thickness between disks, which is equivalent to the size of average proteins, the phenomenon of steric volume exclusion is remarkable in photoreceptor outer segment. This effect is particularly relevant for the light-dependent translocation of signaling proteins such as recoverin, transducin, and arrestin (Najafi and Calvert, 2012; Pearring et al., 2013). However, diffusion alone cannot justify the directionality of phototransduction protein translocation as it would result in equilibration of these soluble proteins throughout the whole cytoplasm of photoreceptors, which is not the case. Consequently, their translocation is the product of the combination of diffusion and a mechanism of light-dependent retention in particular subcellular compartments, which differs from the conventional ciliary trafficking pathway involving the IFT complex (Pearring et al., 2013).

**FIGURE 4:**
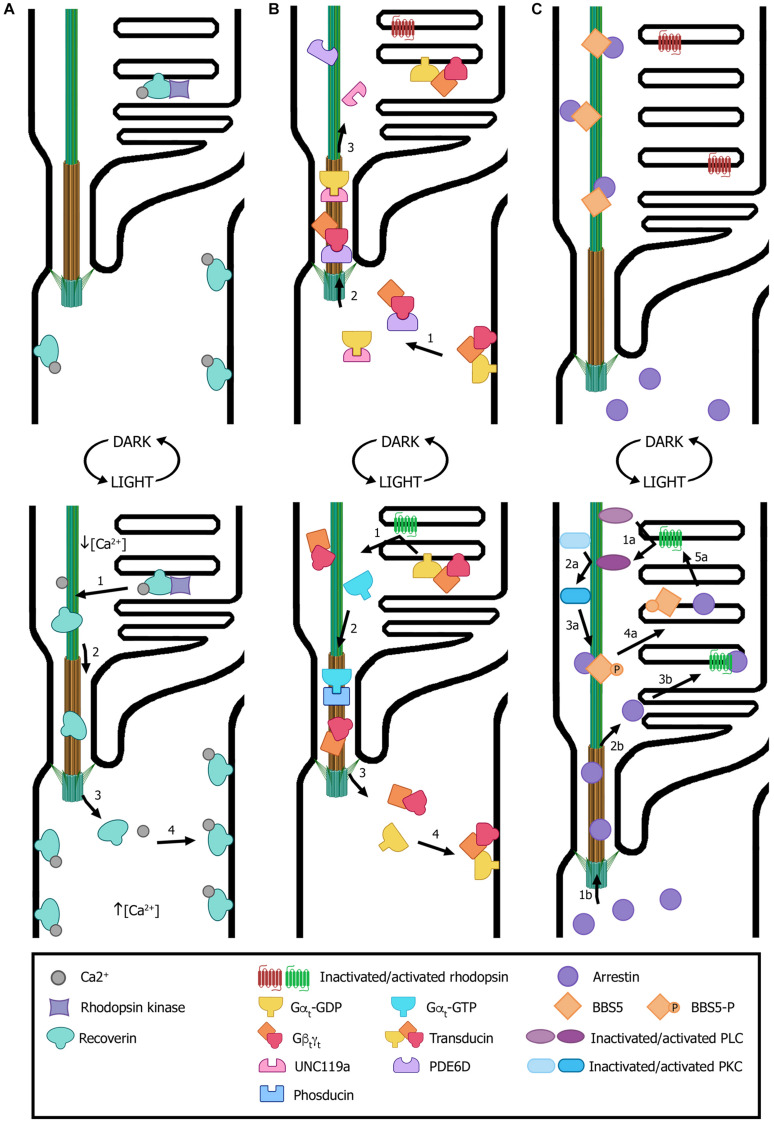
Light-dependent translocation of signaling proteins. **(A)** In dark-adapted rods (top panel), recoverin is bound to Ca^2+^ and distributed along all subcellular compartments; in the outer segment, recoverin sequesters rhodopsin kinase. After light exposure (bottom panel), Ca^2+^ levels decrease in the outer segment and recoverin modifies its conformation to a Ca^2+^-free soluble form. This results in recoverin dissociation from disk membranes and its diffusion from the outer segment through the connecting cilium to other cellular compartments with higher Ca^2+^ concentrations, where it binds the cytoplasmic membrane. **(B)** In dark conditions (top panel), transducin subunits Gα_*t*_-GDP and Gβ_*t*_γ_*t*_ are transported by the trafficking chaperones UNC119a and PDE6D, respectively, to the outer segment. There, the heterotrimer transducin binds to disk membranes. In light conditions (bottom panel), photoexcited rhodopsin induces GPD/GTP exchange in the Gα_*t*_ subunit, resulting in heterotrimer dissociation and diffusion to the inner segment. Gα_*t*_ translocation is assisted by phosducin. Once in the inner segment, GTP is hydrolyzed, causing Gα_*t*_-GdP and Gβ_*t*_γ_*t*_ re-association and binding to the cytoplasmic membrane. **(C)** In dark conditions (top panel), arrestin is mostly found at the inner segment and the small fraction contained within the outer segment is sequestered by BBS5. Upon light exposure (bottom panel), activated arrestin can follow two independent pathways to interact with activated rhodopsin (pathways “a” and “b”). One pathway involves photoexcited rhodopsin interacting and activating phospholipase (PLC), which in turn activates protein kinase C (PKC). Activated PKC then phosphorylates BBS5, resulting in the release of arrestin, which can now diffuse freely and interact with activated rhodopsin (“1a” to “5a” steps). Alternatively, arrestin diffuses from the inner segment into the outer segment and binds to photoexcited rhodopsin (“1b” to “3b” steps). Numbers in each panel indicate the sequence of transport steps. In all images, only selected proteins are represented, for the sake of clarity. (Figure created with BioRender.com).

Recoverin is a Ca^2+^-binding protein with dual functions: in the outer segment it regulates the activity of rhodopsin kinase (RK), whereas in the synaptic terminals it modulates photoreceptor synaptic output (Klenchin et al., 1995; Sampath et al., 2005; Chen et al., 2012). In dark-adapted rods, recoverin is distributed along all subcellular compartments (Figure 4A). Sustained illumination induces its translocation from the outer segment to synaptic terminals, while other compartments remain almost unaltered (Strissel et al., 2005). The proposed mechanism that explains recoverin light-dependent redistribution is that, upon illumination, Ca^2+^ levels decrease in the outer segment and recoverin changes its conformation from the Ca^2+^-bound form – which possesses high membrane affinity – to its soluble Ca^2+^-free configuration. This would result in its dissociation from disk membranes, its diffusion through the connecting cilium and its re-association with membranes in cellular subcompartments where Ca^2+^ concentration is higher (Figure 4A) (Pearring et al., 2013).

Transducin is a G protein that mediates visual signaling between photoexcited rhodopsin (R^∗^) and the photoreceptor phosphodiesterase (PDE6), which is the central effector enzyme in the phototransduction cascade, and helps rods to yield considerably amplified photoresponses (Arshavsky and Wensel, 2013). In the dark, transducin translocation to the outer segment is assisted by the trafficking chaperones UNC119a and PDE6D. There, heterotrimeric transducin binds to disk membranes with high affinity thanks to lipid modifications (Figure 4B) (Constantine et al., 2012). Light exposure causes rhodopsin photoexcitation that binds to transducin and induces its activation by means of the separation of the GTP-bound Gα_*t*_ subunit from the non-dissociable Gβ_*t*_γ_*t*_ subunit. This reduces the membrane affinity of transducin subunits and leads to the dissociation of both Gα_*t*_ and Gβ_*t*_γ_*t*_ from disk membranes. Then, transducin translocation from rod outer segment to other cellular subcompartments is facilitated by dephosphorylated phosducin (Sokolov et al., 2004). Once in the inner segment and synaptic terminals, Gα_*t*_ is converted into the GDP-bound state, associates with Gβ_*t*_γ_*t*_ and the heterotrimer binds to the cytoplasmic membrane (Figure 4B) (Constantine et al., 2012).

Arrestin follows the opposite movement to that of transducin and it contributes to photoresponse deactivation by interacting with R^∗^ and thereby ceasing transducin activation (Broekhuyse et al., 1985). In dark-adapted outer segments, arrestin is mainly found at the inner segment and only a small fraction is contained within the outer segment (Figure 4C). Light-induced translocation to the outer segment is proposed to be triggered by an intracellular signal downstream from the phototransduction cascade, which releases arrestin from its low affinity binding sites in the IS and results in its subsequent equilibration between the photoreceptor IS and outer segment by diffusion (Pearring et al., 2013). Activation of phospholipase C (PLC) and protein kinase C (PKC) downstream of rhodopsin appears to start arrestin translocation (Orisme et al., 2010). Furthermore, arrestin was found to directly interact with BBS5, retaining outer segment arrestin along the axoneme and preventing its function. Upon light exposure, PKC phosphorylates BBS5, which then releases arrestin and enables its free diffusion to bind activated rhodopsin (Figure 4C) (Smith et al., 2013).

## Retinal Ciliopathies and Defects in Ciliary Transport

Defects in ciliary genes can cause dysfunction of the cilium resulting in ciliopathies, a broad group of genetically and phenotypically heterogeneous inherited disorders. Retinal disease is a frequent phenotype due to the highly specialized neurosensory cilium that photoreceptors possess (Table 1). Retinopathies can be developed as a consequence of incorrect ciliogenesis or defective ciliary transport of signaling or structural proteins, and the existence of many causative genes for retinal ciliopathies highlights the diversity of protein functions necessary for normal photoreceptor functioning.

**TABLE 1:** Inherited ciliopathies with retinal involvement^a,b,c^.

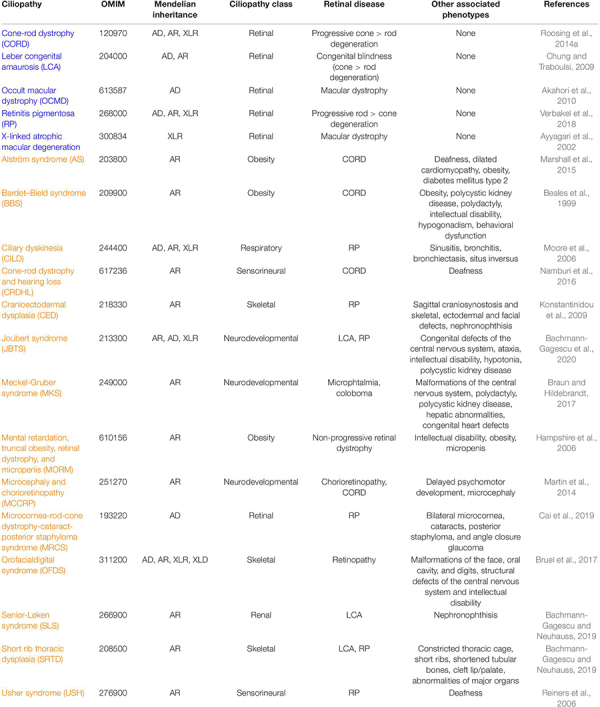

*^*a*^From RetNet and OMIM databases.*

*^*b*^AD, autosomal dominant; AR, autosomal recessive; XLD, X-linked dominant; XLR, X-linked recessive.*

*^*c*^Non-syndromic ciliopathies are in blue, and syndromic ciliopathies are in orange.*

To date, mutations in more than 100 ciliary genes have been associated with retinal dystrophies (Supplementary Table 1 and Figure 2), accounting for almost 25% of retinal disease (Chen et al., 2019). At least, half of the ciliary genes related to retinal disease are involved in ciliary transport, probably due to the high protein demand of photoreceptor outer segments, which requires efficient import and export of proteins. Depending on the function of the gene product, the mutation type, the site of mutation within the gene, and even genetic modifiers, retinal ciliopathies can present different phenotypic manifestations that are classified into two groups: non-syndromic retinal ciliopathies – in which only retinal degeneration is described because functional defects are exclusively confined to photoreceptor cilia – and syndromic retinal ciliopathies – which are accompanied with phenotypes in other tissues due to a broader range of ciliary disruption. In this section, we are going to focus exclusively on genes that are related to ciliary transport.

### Non-syndromic Retinal Ciliopathies

Some mutations in ciliary genes appear to exclusively disrupt photoreceptor function and compromise its survival, thus causing non-syndromic retinal ciliopathies. Depending on the photoreceptor type that is primarily affected, non-syndromic retinal ciliopathies present different clinical manifestations.

#### Retinitis Pigmentosa

Retinitis pigmentosa (RP, MIM 268000) is the most common inherited retinal dystrophy with a worldwide prevalence of 1:4,000 in its non-syndromic form (Verbakel et al., 2018). It is characterized by the primary degeneration of rod photoreceptors and the secondary loss of cone photoreceptors. Consequently, patients initially develop night blindness and difficulty with dark adaptation, which further progress to peripheral and dim light visual defects in dim light and a gradual reduction of central vision when cones start being affected (Verbakel et al., 2018).

Retinitis pigmentosa can follow all patterns of Mendelian inheritance and its severity correlates with its type of inheritance, autosomal dominant RP presenting the best long-term prognosis whereas X-linked RP has the most severe degenerating process (Hamel, 2006; Verbakel et al., 2018).

It has been estimated that ciliary genes represent near 40% of RP genetic causes. Mutations in many of these genes can also result in syndromic ciliopathies with RP as one of the characteristic clinical features, as it is the case in Usher and Bardet–Biedl syndromes (Estrada-Cuzcano et al., 2012b). For instance, mutations in *USH2A* are one of the most frequent causes of autosomal recessive RP but can also result in Usher syndrome (Lenassi et al., 2015). Other important ciliary genes in RP are *RP1*, associated with autosomal dominant RP, and *RPGR* and *RP2*, both causative genes of X-linked RP (Sullivan et al., 1999; Megaw et al., 2015).

*RPGR* is mutated in 70–90% of X-linked RP patients, accounting for 10–20% of all RP cases (Megaw et al., 2015). Mutations in *RPGR* have also been associated with cone-rod dystrophy (CORD) and X-linked atrophic macular degeneration (Ayyagari et al., 2002; Shu et al., 2007). *Rpgr* mutant mice have been generated as a model of X-linked RP and they recapitulate the progressive degeneration of photoreceptor. This phenotype is due to defects in ciliary transport. RPGR-defective mouse retinas exhibit mislocalization of cone opsins and reduced rhodopsin levels in rods, evidencing the role of the connecting cilium protein RPGR in the maintenance of the polarized protein distribution by regulating directional protein transport and/or cargo sorting (Hong et al., 2000; Brunner et al., 2010; Thompson et al., 2012). Interestingly, an early onset cone-rod degeneration was observed in the mutant individuals of the BALB/c line, which suggests the existence of genetic modifiers. This mutant strain is a model for cone-rod degeneration due to *RPGR* mutations (Brunner et al., 2010).

*Rp2* knockout mice also mimic X-linked RP. Their photoreceptors have a constantly high concentration of hyperactive ARL3-GTP as RP2 is not present to induce ARL3 GTPase activity and, consequently, its inactivation. The hyperactive ARL3-GTP promotes PDE6D closed conformation, preventing the trafficking chaperone to interact with its cargo and, thereby, hampering ciliary import of lipidated proteins such as GRK1 and PDE6 (Baehr, 2014; Zhang et al., 2015). Additionally, it has been recently proposed that in cones, RP2 may modulate the availability of proteins that are implicated in shedding of cone outer segments by controlling their ciliary import (Li et al., 2019).

#### Cone-Rod Dystrophy (CORD, MIM 120970)

Cone-rod dystrophy (MIM 120970) is a progressive cone degenerating disorder (prevalence of 1:30,000–1:40,000, Estrada-Cuzcano et al., 2012b). It is characterized by the primary loss of cone photoreceptors, closely followed by or simultaneous with rod degeneration (Hamel, 2007). Clinical symptoms become noticeable during primary school years and comprise photo aversion, diminished visual acuity, color vision defects and reduced sensitivity of the central visual field. As rod photoreceptors become also affected, patients may also display night blindness and loss of peripheral vision (Estrada-Cuzcano et al., 2012b). CORD can follow all types of Mendelian inheritance and appear as an isolated disorder or forming part of a syndromic case (Roosing et al., 2014b). So far, mutations in 14 ciliary genes have been identified in CORD patients, the most relevant ones being *C8orf37*, *RPGR*, and *RPGRIP1*, and many of them are involved in ciliary transport (Gill et al., 2019). In this context, it is remarkable that one *Rpgr* mutation has been reported to cause different retinal dystrophies (either RP or CORD) depending on the mouse genetic background, thus implying the existence of specific genetic modifiers with an impact on ciliary transport in different photoreceptor cell types (Brunner et al., 2010).

#### Leber Congenital Amaurosis

Leber congenital amaurosis (LCA, MIM 204000) is the most severe retinal dystrophy, since patients suffering from profound vision impairment before their first year of age (prevalence 1:30,000 and 1:81,000) and it generally follows an autosomal recessive Mendelian pattern of inheritance (Koenekoop, 2004; den Hollander et al., 2008).

Based on genetics and ocular phenotypes, LCA is classified in thirteen different types. Mutations in ciliary genes represent around 25% of disease-causing genes. In fact, mutations in *CEP290*, a key element of the ciliary diffusion barrier at the transition zone, are the most common causes of LCA in some populations (Chung and Traboulsi, 2009; Estrada-Cuzcano et al., 2012b). *LCA5* is also an important LCA causative gene and it encodes the connecting cilium protein lebercilin, which participates in IFT transport. Leberlicin has been reported to interact with IFT complexes and when mutated, ciliary trafficking is perturbed (den Hollander et al., 2007; Boldt et al., 2011).

In *Lca5* mutant mice, photoreceptors display partial mislocalization of opsins and reduction of light-induced translocation of arrestin to the outer segments. Defective ciliary trafficking due to the inability of lebercilin to interact with the IFT machinery results in early onset photoreceptor degeneration in *Lca5* mutant mice, mimicking the LCA phenotype (Boldt et al., 2011). In a recent study, *lca5^–/–^* zebrafish photoreceptors presented an abnormal distribution of Ift88, which is in agreement with lebercilin interacting with the IFT machinery (Qu et al., 2019).

Mutations in *RPGRIP1* can cause RP, CORD, and LCA in humans. In mouse photoreceptors, RPGRIP1 localizes predominantly to the connecting cilium and it is required for rod outer segment development and ciliary targeting of RPGR, NPHP4, and SDCCAG8, all of them associated with retinal ciliopathies (Won et al., 2009; Patil et al., 2012). *Rpgrip1* mutant mice show deficient rod outer segment morphogenesis whereas cone outer segments initially form but degenerate rapidly, which suggests that the role of RPGRIP1 in outer segment development is different between cones and rods (Won et al., 2009). *rpgrip1* null zebrafish exhibit similar phenotypes to those observed in juvenile RP patients and LCA. *rpgrip^–/–^* photoreceptors display mislocalization of opsins and, interestingly, also of Rab8, a key player in rhodopsin-carrying vesicle transport. Thus, at least in zebrafish, Rpgrip1 is also necessary for Rab8 localization to the cilium and, consequently, rhodopsin transport (Raghupathy et al., 2017).

Mutations in *IFT38*, a component of the IFT-B complex, are also associated with LCA in humans. *Ift38* knockout mice are embryonically lethal due to loss of primary cilia, which resulted in disruption oft Sonic hedgehog signaling during embryonic development (Pasek et al., 2012). In zebrafish, *ift38* mutants exhibit normal development of photoreceptor cells, followed by progressive photoreceptor degeneration caused by alterations in ciliogenesis, a phenotype that resembles the LCA phenotype in humans much more than the embryonic lethality in mutant mice (Lee et al., 2014).

#### Macular Dystrophy

Mutations in two ciliary genes (*RPGR* and *RP1L1*), both localized in the connecting cilium, have been described in patients with macular dystrophies.

RP1L1 is a structural component of the ciliary axoneme required for proper organization and structure of the photoreceptor outer segments (Yamashita et al., 2009). Defects in *RP1L1* cause occult macular dystrophy (OCMD, MIM 613587), an autosomal dominant macular dystrophy. OCMD is characterized by a gradual decrease in visual acuity due to the progressive loss of macular function even though ophthalmoscopic appearance is normal (Akahori et al., 2010; Fujinami et al., 2016).

Regarding *RPGR*, 3% of mutations in this ciliary gene have been associated with X-linked atrophic macular degeneration (MIM 300834) (Ayyagari et al., 2002; Shu et al., 2007). Patients with X-linked atrophic macular degeneration show progressive central vision loss with minimal peripheral visual impairment even in advancing age (Ayyagari et al., 2002).

### Syndromic Retinal Ciliopathies

Retinal degeneration is a frequent phenotype of an extensive variety of syndromic ciliopathies in which multiple cell types and tissues are affected due to ciliary dysfunction. Many ciliopathies have overlapping phenotypes and there is no clear genotype–phenotype correlation in most of them, as mutations in the same gene can cause different syndromes. Therefore, it exists an underlying complexity between a ciliary gene and associated ciliopathies.

#### Alström Syndrome

Alström syndrome (AS, MIM 203800) is a rare, autosomal recessive disorder (prevalence 1:1,000,000–9:1,000,000) caused by mutations in *ALMS1*, a broadly expressed gene whose gene product is a component of the centrosome and has been proposed to be involved in ciliary transport and cell cycle regulation (Collin et al., 2002; Hearn et al., 2002, 2005; Marshall et al., 2015; Hearn, 2019). AS patients present progressive vision loss due to CORD, sensorineural hearing loss, childhood obesity and diabetes mellitus type 2 (Marshall et al., 2005, 2007). Even though there is one causative gene, the ocular phenotype is quite variable and different ocular cell types may be affected, which suggests that cilia are crucial for multiple aspects of visual function (May-Simera et al., 2017).

ALMS1 seems to have multiple functions, which would explain the phenotypic complexity of AS. *Alms1* mutant mice mimicked AS patients’ phenotypes. In ALMS1-deficient photoreceptors, rhodopsin partly mislocalizes and vesicles accumulate in the inner segment, which indicates that ALMS1 participates in the shift from vesicular transport to intraciliary trafficking at the base of the connecting cilium (Collin et al., 2005; Hearn, 2019).

#### Bardet–Biedl Syndrome

Bardet–Biedl syndrome (BBS, MIM 209900) is an autosomal recessive cilia-related disorder characterized by CORD, renal abnormalities, obesity, polydactyly, hypogonadism, intellectual disability, and behavioral dysfunction (Beales et al., 1999). It has a prevalence of approximately 1:100,000 in Europe and North America, but it is more frequent in particular isolated communities (Forsythe et al., 2018). BBS is caused by mutations in more than 20 identified genes that lead to defects in the assembly, composition or localization of the BBSome, thus resulting in the loss or anomalous accumulation of ciliary proteins due to deficient ciliary trafficking (Nachury et al., 2007; Forsythe et al., 2018; Liu and Lechtreck, 2018).

Most BBS model organisms that present retinal phenotypes show aberrant localization of rhodopsin to the inner segment, outer segment disorganization and progressive photoreceptor degeneration. Animal models deficient for any BBS protein share a similar retinal degeneration phenotype. As shown in a *Lztfl1* mutant mouse model, this photoreceptor degeneration is probably due to the mislocalization and accumulation of non-outer segment proteins in the outer segment, pointing to the loss of compartmentalization, rather than defects in protein import to the outer segment. Therefore, these results further support that the BBSome is a ciliary gatekeeper or participates in retrograde protein trafficking (Datta et al., 2015).

#### Joubert Syndrome

Joubert syndrome (JBTS, MIM 213300), a recessive developmental disorder with a high clinical and genetic variability, is caused by mutations in more than 30 genes (prevalence 1:50,000–1:100,000). It is characterized by congenital defects of the central nervous system, ataxia, intellectual disability, hypotonia, neonatal breathing difficulties and abnormal eye movements. Less frequently, patients may present in addition retinal degeneration and renal anomalies (Dilan et al., 2019; Bachmann-Gagescu et al., 2020). The molecular function of JBTS proteins is diverse, some proteins localizing to the ciliary axoneme, others to the transition zone or the basal body, but all of them are involved in ciliogenesis or ciliary function (Hua et al., 2017). Most mutations causing JBTS affect genes that belong to the MKS module of the transition zone, including *TMEM67*, *CC2D2A*, and *AHI1* (Bachmann-Gagescu et al., 2015a; Vilboux et al., 2017).

*Ahi1* null mice show cilium-specific vesicular defects probably caused by a reduction of Rab8a levels in photoreceptor cells, indicating that Ahi1 is important for its stabilization. *Ahi1^–/–^* mice present retinal degeneration that is consistent with the ocular phenotype of JBTS patients (Westfall et al., 2010). Animal models of *TMEM67* and *CC2D2A* will be explained in the following section as the manifested phenotypes are more in association with MKS.

#### Meckel–Gruber Syndrome

Meckel–Gruber syndrome (MKS, MIM 249000), the human ciliopathy presenting the most severe clinical manifestations, is an autosomal recessive developmental condition that frequently leads to embryonic or perinatal lethality due to dysfunction of primary cilia during early embryogenesis. It shows an extensive clinical heterogeneity and some of the main symptoms include malformations of the central nervous system, cystic renal disease, hepatic abnormalities, congenital heart defects, polydactyly and ocular alterations (MacRae et al., 1972; Braun and Hildebrandt, 2017). Currently, 13 different causative genes have been identified and most of the proteins that are associated with MKS are found at the transition zone (Garcia-Gonzalo et al., 2011; Sang et al., 2011; Szymanska et al., 2015). Mutations in *TMEM67* are responsible for 16% of all MKS cases, being the most common cause of this ciliopathy, followed by *MKS1* mutations, which account for 7% of MKS cases (Hartill et al., 2017). Both MKS1 and TMEM67, which are reciprocal interactors, have been found to be required for ciliogenesis and work together with the BBSome to assist ciliary trafficking of particular transmembrane receptors (Dawe et al., 2007; Goetz et al., 2017). *Tmem67* mouse mutants display abnormal photoreceptor outer segments that lack rhodopsin due to alterations in the movement of ciliary proteins through the connecting cilium. TMEM67-deficient photoreceptor cells degenerate early and rapidly and mutant mice develop features similar to the retinal phenotype in MKS patients (Collin et al., 2012).

Mutations in *CC2D2A* can cause non-syndromic RP and CORD or syndromic ciliopathies such as JBTS, MKS and COACH. *Cc2d2a^–/–^* mice mimic Meckel syndrome phenotype, being embryonically lethal and exhibiting multiorgan defects due to defective ciliogenesis (Veleri et al., 2014). In *cc2d2a^–/–^* zebrafish, photoreceptors suffer from cilium-specific vesicle fusion defects due to the loss of SNAP25, Syntaxin3 and Exoc4, resulting in the accumulation of vesicles carrying opsin. In contrast to *Cc2d2a* knockout mice, *cc2d2a^–/–^* fish do not present defects in ciliogenesis (Ojeda Naharros et al., 2017).

#### Orofaciodigital Syndrome

Orofaciodigital syndrome (OFDS, 311200) comprises a group of conditions characterized by malformations of the face, oral cavity and digits. Some patients also show structural defects of the central nervous system and intellectual disability (Adams et al., 2007). OFDS type 1 (OFD1, MIM 311200) is an X-linked dominant ciliopathy caused by mutations in *OFD1* gene, involved in ciliogenesis and cilia length regulation, and it is distinguished from other OFDS subtypes by adult-onset cystic kidney disease in addition to its inheritance pattern (Ferrante et al., 2001; Singla et al., 2010). Interestingly, a deep intronic mutation in *OFD1* has been identified as causative of non-syndromic RP (Webb et al., 2012).

#### Senior–Løken Syndrome

Senior–Løken syndrome (SLS, MIM 266900), or renal–retinal dysplasia, refers to the syndromic association of juvenile NPHP (MIM 256100), an autosomal recessive cystic kidney disease with early onset retinal involvement (Braun and Hildebrandt, 2017). Usually, all patients develop ocular alterations by the age of 10 years, among them RP, which is the most frequent extra-renal phenotype of NPHP-related ciliopathies (Adams et al., 2007). As in MKS and JBTS, most SLS proteins are found at the ciliary transition zone (Ronquillo et al., 2012). Mutations in *NPHP5* are the most common cause of SLS (Otto et al., 2005). NPHP5 is a centrosomal and transition zone protein which forms a distinct complex with CEP290, also reported to be mutated in SLS patients (Sayer et al., 2006; Barbelanne et al., 2013). Together, they regulate BBsome integrity and trafficking of the complex and its cargo (Barbelanne et al., 2015).

In *Nphp5^–/–^* mice, photoreceptors do not fully develop the connecting cilium and are unable to form outer segments, thus resulting in complete photoreceptor degeneration at 1 month of age (Ronquillo et al., 2016). Absence of NPHP5 results in mislocalization of BBS2 and BBS5, consequently compromising the integrity of the BBSome in hTert-RPE1 cells. Since the BBSome is dysfunctional, the IFT-dependent elongation of the axoneme is also perturbed, hence the late ciliogenesis alterations (Barbelanne et al., 2015; Ronquillo et al., 2016).

The retinal phenotype in *Nphp5* mutants resembles LCA and is compatible with SLS. However, other clinical manifestations characteristic of SLS, such as kidney disease, are absent in *Nphp5^–/–^* mice, thus not fully recapitulating this human disorder. This phenomenon has also been observed in other knockout mouse models of NPHP1, NPHP4, and NPHP6, where the only conserved disease phenotype was retinal degeneration (Ronquillo et al., 2016).

#### Short-Rib Thoracic Dysplasia

Short-rib thoracic dysplasia (SRTD, MIM 208500), with or without polydactyly, comprises a group of autosomal recessive skeletal ciliopathies characterized by short ribs, shortened tubular bones and constricted thoracic cage, which leads to respiratory insufficiency that usually causes death in new-borns. Other non-skeletal phenotypes are cleft lip/palate, retinal degeneration and abnormalities of major organs including the brain, heart, liver, kidneys, pancreas, intestines, and genitalia. It encompasses Jeune syndrome or asphyxiating thoracic dystrophy, short rib-polydactyly syndrome, Mainzer-Saldino syndrome and Ellis-van Creveld syndrome (Huber and Cormier-Daire, 2012). Many SRTD patients present mutations in genes encoding elements of the IFT machinery. Approximately, 17 causative genes have been identified and, interestingly, mutations in some of them have also been associated with non-syndromic RP, as it is the case with *IFT172* and *IFT140*, components of the IFT-B and IFT-A complexes, respectively (Bujakowska et al., 2015, 2017; Xu et al., 2015; Bifari et al., 2016).

Several animal models have been generated to study the pathological mechanisms due to defects in IFT proteins. In zebrafish, mutations in *ift172* result in mislocalization of opsins and retinal degeneration. Photoreceptors are unable to form outer segments suggesting that *ift172* plays a crucial role in the initiation of outer segment formation (Sukumaran and Perkins, 2009). In rod-specific *Ift172* knockout mice, loss of IFT172 causes rapid degeneration of the retina, with abnormal localization of outer segment proteins such as rhodopsin, IFT139 and RP1, overall indicating alterations in ciliary trafficking that seriously compromise photoreceptor survival (Gupta et al., 2018).

#### Usher Syndrome

Usher syndrome (USH, MIM 276900) is a group of autosomal recessive disorders characterized by congenital, bilateral deafness and progressive RP. It is divided into three groups of decreasing severity and later progression of the symptoms and onset time: USH1, USH2, and USH3 (Reiners et al., 2006).

USH is the most common cause of combined hereditary deafness and blindness and it is caused by genes encoding proteins belonging to different functional families. Consequently, USH proteins have miscellaneous molecular functions, including actin-based transport, cellular adhesion, and signal transduction (May-Simera et al., 2017). Some USH proteins relevant in the retina are Myosin VII, which is the most studied USH protein in this tissue, and the periciliary membrane USH2 complex, consisting of USH2A, USH2C, and USH2D proteins (Mathur and Yang, 2015). Although USH mouse models faithfully recapitulate hearing disease, they do not mimic the retinal phenotype observed in USH patients, probably due to species-specific differences of the USH complex. In fact, only mice with mutations in *Ush1c*, *Ush2a*, or *Ush2d* have shown retinal degeneration (El-Amraoui and Petit, 2014).

## Molecular and Genetic Complexity Underlying Retinal Ciliopathies

The molecular cause of ciliopathies is remarkably complex and, although cilia are rather ubiquitously found in mammalian cells, not all mutations in ciliary genes that cause retinal degeneration are also associated with pleiotropic pathologies in other ciliated organs and vice versa (Supplementary Table 1).

### Transcriptional Regulation in Non-syndromic Retinal Ciliopathies

The adult human retina presents a great transcript diversity and part of this heterogenity can be explained by the “switch-like” splicing pattern that photoreceptors display, resulting in higher inclusion levels of exons that are not found in transcripts outside the retina (Farkas et al., 2013; Murphy et al., 2016). Interestingly, many genes associated with retinal ciliopathies have retina-specific isoforms, among them transcripts encoding photoreceptor ciliary proteins (Mellough et al., 2019). Whether these retina-specific isoforms are also involved in ciliary transport or in other cellular or ciliary pathways remains to be investigated.

A very good example is *TTC8/BBS8*, which is mutated in BBS and presents a photoreceptor-specific isoform that includes a cassette exon, named exon 2a. When this exon is mutated, patients present non-syndromic RP. Other cell types do not express this isoform, and therefore, remain unaffected (Riazuddin et al., 2010; Murphy et al., 2015).

Mutations in *ARL6/BBS3* result in BBS syndrome. However, it presents a retina-specific splice variant, named *BBS3L*, originated by the inclusion of an extra 13 bp exon near the 3′ end, which shifts the open reading frame, producing a different C-terminus of the protein. The *BBS3L* transcript is specifically needed for correct retinal function and organization and, consequently, patients with mutations exclusively affecting this isoform would be expected to develop non-syndromic RP (Pretorius et al., 2010).

*RPGR* also has a retina-specific transcript, which includes a large 3′ terminal exon (ORF15) that is mutated in 60% of X-linked RP patients, which highlights the functional relevance of RPGR in the retina (Vervoort et al., 2000; Megaw et al., 2015).

Secondary ciliary dysfunction can also arise as a consequence of genetic variants in spliceosomal proteins, but the effect of splicing on ciliogenesis and ciliary function genes is out of the scope of this review.

### Post-translational Regulation in Retinal Ciliopathies and Ciliogenesis

Ciliary defects can also be caused by mutations in genes not directly involved in ciliary function, structure or biogenesis but in its regulation. More than 100 proteins involved in sensory cilia have been found to be controlled by ubiquitin and small ubiquitin-like modifier (SUMO) post-translational modifications, pointing that genes belonging to the ubiquitin-proteasome system (UPS) might contribute to ciliary structure and function in photoreceptors (Toulis and Marfany, 2020). Recently, *Atxn3* loss of function in zebrafish and mouse models has been reported to cause retinal ciliogenesis dysregulation and photoreceptor dysfunction (Toulis et al., 2020).

So far, mutations in two UPS genes, both encoding E3 ubiquitin ligases, have been unequivocally determined as causative genes for inherited retinal ciliopathies in humans: *TOPORS*, causative of non-syndromic autosomal dominant RP, and *TRIM32/BBS11*, causative of syndromic autosomal recessive BBS (Chiang et al., 2006; Chakarova et al., 2011). These proteins localize to the basal body. While TRIM32 regulates the stability of NPHP7 (Ramachandran et al., 2014), the retinal substrate of TOPORS is yet to be determined although physical association with dynactin subunits suggests that it might regulate ciliary transport (Chakarova et al., 2011).

Interestingly, together with *RP1*, *TOPORS* is one of the only two ciliary genes associated with autosomal dominant RP (Sullivan et al., 1999; Chakarova et al., 2007). Mutations in *TOPORS* cause premature protein truncation and, therefore, haploinsufficiency has been proposed as the pathogenic cause. As ciliary proteins work in multiprotein complexes, haploinsufficiency due to the disruption of one allele might be partially compensated by a different member of the complex. Therefore, for most proteins of the complex, phenotypes would be recessive and require mutations of the two alleles. Conversely, mutations in different genes of the same complex (e.g., BBSome and NPHP-JBTS-MKS complexes) may cause overlapping or highly similar phenotypes (Estrada-Cuzcano et al., 2012b). However, these two ciliary proteins seem to be particularly critical in photoreceptors as defects in only one allele highly compromise retinal function while other ciliated organs remain unaffected.

### Ciliopathy Severity by Different Type or Combination of Genetic Mutations

In some cases disease severity can be explained by the primary disease-causing mutation or the position of a mutation within the gene, resulting in null, hypomorph or dominant-negative alleles that alter functional modules in different ways and even in different cell types. According to this, it would be expected that loss-of-function variants had a higher impact on the retina – as a highly sensitive tissue – than in other ciliated tissues, explaining why mutations in ciliary genes can cause non-syndromic retinal ciliopathies. On the other hand, gain-of-function variants or dominant-negative alleles would greatly disrupt ciliary function and result in cilia dysfunction in most ciliated tissues. However, these expectations are not fulfilled in many cases, and therefore, establishing genotype-phenotype correlations in retinal ciliopathies is often difficult. Nevertheless, ciliopathy genes are good candidates to cause inherited retinopathies and, currently, multigene panels for molecular diagnosis of inherited retinal dystrophies include ciliopathy genes in the mutational screening.

Furthermore, animal models have demonstrated that combinations of ciliopathy alleles are not necessarily associated with more severe phenotypes, revealing that ciliary proteins establish intricate relationships. For example, mouse *Dync2h1* mutants had milder phenotypes when harboring heterozygous loss of *Ift172.* Dync2h1 is involved in retrograde IFT while Ift172 participates in anterograde IFT, thus suggesting that the equilibrium was partially restored due to the neutralization or compensation of two opposite dysfunctions. However, knockdown of *Ift122*, which is part of the IFT-A complex, was also able to rescue the *Dync2h1* phenotype, reinforcing the idea of complexity in the interactions of ciliary proteins (Ocbina et al., 2011). Another study also found that heterozygous disruption of *Mkks*, a BBS chaperonin protein, in *Cep290^*rd*16/rd16^* mice ameliorated the sensory cilia defects, and vice versa (Rachel et al., 2012).

Indeed, the same mutation in the same gene can result in a heterogeneity of disorders, from different syndromic ciliopathies to isolated forms of retinal ciliopathies, implying there are more players involved in the phenotype. Cases in which *cis*-acting regulators of expression or *trans*-acting epistatic modifier alleles may be the origin of phenotypic variation are reported for *BBS1* and *IFT172*. The *BBS1* p.M390R variant was found in homozygosis in RP and BBS patients as well as in unaffected parents of BBS patients and in a control proband. The study proposed that different promoter or enhancer polymorphisms could lead to distinct levels of transcriptional expression of the mutated alleles, thus resulting in less or more severe phenotypes (Estrada-Cuzcano et al., 2012a). Additionally, epistatic effects of other alleles were also proposed to modulate the phenotypes associated with *IFT172* mutations, as the functional analysis could only partially explain milder phenotypes (Bujakowska et al., 2015).

## Concluding Remarks

In spite of sharing common molecular mechanisms with primary cilia, the photoreceptor neurosensory cilium undergoes a highly refined specialization. Ciliary protein trafficking is essential for ciliogenesis and photoreceptor function, as reflected by the existence of non-syndromic and syndromic retinal ciliopathies associated with mutations in proteins involved in ciliary transport. Indeed, the cilium is a key structure in photoreceptor function and survival and, consequently, all ciliary genes and their regulators are potentially plausible candidates for non-syndromic and syndromic retinal dystrophies. It is possible that non-syndromic mutations in syndrome-associated genes have not been reported yet, and vice versa. Further elucidation of common and photoreceptor-specific ciliary protein networks and functional interactions are needed to understand the pleiotropy of phenotypes that arise from mutations in ciliary genes, which alter ciliary structure and/or function. Deciding whether therapy should target rearing ciliary structures, reinstating ciliary trafficking, or both will be key to devise efficient treatments for retinal ciliopathies.

## Author Contributions

LS-B and VT wrote a draft and contributed to figures. GM provided the idea, the funding, and has supervised the work. All the authors have contributed to the writing and revision of the manuscript.

## Conflict of Interest

The authors declare that the research was conducted in the absence of any commercial or financial relationships that could be construed as a potential conflict of interest.
