# Supplementary material for: On the Wrong Track: Alterations of Ciliary Transport in Inherited Retinal Dystrophies
Source: Front Cell Dev Biol. 2021 Mar 5;9:623734. doi: 10.3389/fcell.2021.623734 (PMC7973215; doi:10.3389/fcell.2021.623734)
Supplement: Supplementary file 1 [file Table_1.pdf]

TABLE 1. Comprehensive list of ciliary genes associated with retinal dystrophies in syndromic and non-syndromic disorders[a,b,c].

	Non-syndromic retinal ciliopathies				Syndromic ciliopathies with retinal degeneration														Other ciliopathies without retinal degeneration						
	RP	CORD	LCA	MD	AS	BBS	CED	CILD	CRDHL	JBTS	MCCRP	MKS	MORM	MRCS	OFDS	SLS	SRTD	USH	COACH	LGMD	MKKS	NPHP	NSD	SGBS	References
AHI1	✓									✓															(Nguyen et al., 2017)
AIPL1		✓	✓																						(Sacristan-Reviriego et al., 2020)
ALMS1		✓	✓		✓																				(Xu et al., 2016)
ARL13B										✓															(Cantagrel et al., 2008)
ARL2														✓											(Cai et al., 2019)
ARL2BP	✓																								(Davidson et al., 2013)
ARL3	✓	✓								✓															(Sheikh et al., 2019)
ARL6	✓					✓																			(Zenteno et al., 2020)
ARMC9										✓															(Van De Weghe et al., 2017)
ARSG																		✓							(Abad-Morales et al., 2020)
B9D1										✓		✓													(Romani et al., 2014)
B9D2										✓		✓													(Radhakrishnan et al., 2019)
BBS1	✓					✓				✓															(Estrada-Cuzcano et al., 2012a; Suzuki et al., 2016)
BBS10						✓																			(Suspitsin and Imyanitov, 2016)
BBS12						✓																			(Suspitsin and Imyanitov, 2016)
BBS18						✓																			(Suspitsin and Imyanitov, 2016)
BBS2	✓					✓																			(Suspitsin and Imyanitov, 2016)

	Non-syndromic retinal ciliopathies				Syndromic ciliopathies with retinal degeneration														Other ciliopathies without retinal degeneration						
	RP	CORD	LCA	MD	AS	BBS	CED	CILD	CRDHL	JBTS	MCCRP	MKS	MORM	MRCS	OFDS	SLS	SRTD	USH	COACH	LGMD	MKKS	NPHP	NSD	SGBS	References
BBS4						✓																			(Suspitsin and Imyanitov, 2016)f
BBS5						✓																			(Suspitsin and Imyanitov, 2016)
BBS7						✓																			(Suspitsin and Imyanitov, 2016)
BBS8	✓					✓																			(Suspitsin and Imyanitov, 2016)
BBS9						✓																			(Suspitsin and Imyanitov, 2016)
C2orf71	✓	✓																							(Corral-Serrano et al., 2018)
C8orf37	✓	✓				✓																			(Tatour and Ben-yosef, 2020)
CC2D2A	✓									✓		✓							✓						(Méjécase et al., 2019)
CDH23																		✓					✓		(Whatley et al., 2020)
CEP104										✓															(Bachmann-Gagescu et al., 2020)
CEP120										✓							✓								(Bachmann-Gagescu et al., 2020)
CEP164																✓						✓			(Chaki et al., 2012)
CEP19						✓																			(Kanie et al., 2017)
CEP250	✓								✓									✓							(Kubota et al., 2018)
CEP290	✓	✓	✓			✓				✓		✓				✓						✓			(Rachel et al., 2012; Roosing et al., 2017)
CEP41										✓															(Bachmann-Gagescu et al., 2020)
CEP78		✓							✓																(Namburi et al., 2016; Gill et al., 2019)

	Non-syndromic retinal ciliopathies				Syndromic ciliopathies with retinal degeneration														Other ciliopathies without retinal degeneration						
	RP	CORD	LCA	MD	AS	BBS	CED	CILD	CRDHL	JBTS	MCCRP	MKS	MORM	MRCS	OFDS	SLS	SRTD	USH	COACH	LGMD	MKKS	NPHP	NSD	SGBS	References
CEP83										✓												✓			(Braun and Hildebrandt, 2017)
CLRN1	✓																	✓							(Tatour and Ben-yosef, 2020)
CSPP1										✓															(Bachmann-Gagescu et al., 2020)
DYNC2H1	✓																✓								(Vig et al., 2020)
FAM161A	✓																								(Bachmann-Gagescu and Neuhauss, 2019)
FSCN2	✓																								(Chen et al., 2019)
IFT139																	✓					✓			(Niwa, 2016)
IFT140	✓		✓														✓								(Xu et al., 2015)
IFT144							✓									✓	✓					✓			(Braun and Hildebrandt, 2017)
IFT172	✓					✓				✓					✓		✓								(Yamada et al., 2019)
IFT27						✓																			(Suspitsin and Imyanitov, 2016)
IFT38			✓																						(Soens et al., 2016)
IFT43	✓						✓										✓								(Biswas et al., 2017)
IFT52							✓										✓								(Girisha et al., 2016; Chen et al., 2018)
IFT54							✓									✓	✓								(Bizet et al., 2015; Zhang et al., 2018)
IFT80																	✓								(Zhang et al., 2018)
IFT81		✓															✓								(Duran et al., 2016; Dharmat et al., 2017)

	Non-syndromic retinal ciliopathies				Syndromic ciliopathies with retinal degeneration														Other ciliopathies without retinal degeneration						
	RP	CORD	LCA	MD	AS	BBS	CED	CILD	CRDHL	JBTS	MCCRP	MKS	MORM	MRCS	OFDS	SLS	SRTD	USH	COACH	LGMD	MKKS	NPHP	NSD	SGBS	References
INPP5E			✓							✓			✓												(Wang and Deretic, 2014; Fansa et al., 2016)
INVS																✓						✓			(O'Toole et al., 2006)
KCNJ13			✓																						(Sergouniotis et al., 2011)
KIAA0556										✓															(Bachmann-Gagescu et al., 2020)
KIAA0586										✓							✓								(Bachmann-Gagescu et al., 2020)
KIAA1549	✓																								(De Bruijn et al., 2018)
KIF3B															✓										(Cogné et al., 2020)
KIF7										✓															(Bachmann-Gagescu et al., 2020)
KIZ	✓																								(El Shamieh et al., 2014)
LCA5	✓	✓	✓																						(Chen et al., 2016)
LZTFL1						✓																			(Suspitsin and Imyanitov, 2016)
MAK	✓																								(Gray et al., 2018)
MKKS						✓															✓				(Suspitsin and Imyanitov, 2016)
MKS1						✓				✓		✓													(Bader et al., 2016)
MYO7A			✓															✓					✓		(Wang et al., 2011; Whatley et al., 2020)
NEK2	✓																								(Nishiguchi et al., 2013)
NEK8										✓		✓							✓			✓			(Ronquillo et al., 2012)
NPHP1						✓				✓						✓						✓			(Suspitsin and Imyanitov, 2016)

	Non-syndromic retinal ciliopathies				Syndromic ciliopathies with retinal degeneration														Other ciliopathies without retinal degeneration						
	RP	CORD	LCA	MD	AS	BBS	CED	CILD	CRDHL	JBTS	MCCRP	MKS	MORM	MRCS	OFDS	SLS	SRTD	USH	COACH	LGMD	MKKS	NPHP	NSD	SGBS	References
NPHP3												✓				✓						✓			(Luo and Tao, 2018)
NPHP4																✓						✓			(Luo and Tao, 2018)
NPHP5	✓		✓													✓						✓			(Tong et al., 2013; Luo and Tao, 2018)
OFD1	✓									✓					✓									✓	(Franco and Thauvin-Robinet, 2016)
PCDH15																		✓					✓		(Whatley et al., 2020)
PDE6D										✓															(Bachmann-Gagescu et al., 2020)
PDZD7																		✓					✓		(Booth et al., 2015)
PLK4											✓														(Martin et al., 2014)
POC1B		✓								✓															(Beck et al., 2014)
RAB28		✓																							(Gill et al., 2019)
RP1	✓			✓																					(Riera et al., 2020)
RP1L1	✓			✓																					(Noel and MacDonald, 2020)
RP2	✓																								(Zhang et al., 2015)
RPGR	✓	✓		✓				✓																	(Shu et al., 2007)
RPGRIP1	✓	✓	✓																						(Raghupathy et al., 2017)
RPGRIP1L	✓		✓			✓				✓		✓							✓			✓			(Wiegering et al., 2018)
SCAPER	✓																								(Jauregui et al., 2019)
SDCCAG8						✓										✓									(Suspitsin and Imyanitov, 2016)
SPATA7	✓		✓																						(Mackay et al., 2011)

	Non-syndromic retinal ciliopathies				Syndromic ciliopathies with retinal degeneration														Other ciliopathies without retinal degeneration						
	RP	CORD	LCA	MD	AS	BBS	CED	CILD	CRDHL	JBTS	MCCRP	MKS	MORM	MRCS	OFDS	SLS	SRTD	USH	COACH	LGMD	MKKS	NPHP	NSD	SGBS	References
TBC1D32															✓										(Franco and Thauvin-Robinet, 2016)
TMEM107										✓		✓			✓										(Lambacher et al., 2016)
TMEM138										✓															(Bachmann-Gagescu et al., 2020)
TMEM216										✓		✓													(Valente et al., 2010)
TMEM218										✓		✓													(Van De Weghe et al., 2021)
TMEM231										✓		✓			✓										(Roberson et al., 2015)
TMEM237										✓															(Bachmann-Gagescu et al., 2020)
TMEM67						✓				✓		✓							✓			✓			(Radhakrishnan et al., 2019)
TOPORS	✓																								(Chakarova et al., 2007)
TRIM32						✓														✓					(Suspitsin and Imyanitov, 2016)
TTLL5		✓																							(Gill et al., 2019)
TUBB4B			✓																						(Luscan et al., 2017)
TUBGCP4											✓														(Scheidecker et al., 2015)
TULP1	✓		✓																						(Jacobson et al., 2014)
UNC119		✓								✓															(Kobayashi et al., 2000)
USH1C																		✓					✓		(Whatley et al., 2020)
USH1G																		✓					✓		(Whatley et al., 2020)
USH2A	✓																	✓							(Whatley et al., 2020)
USH2C																		✓							(Whatley et al., 2020)
USH2D																		✓					✓		(Whatley et al., 2020)

	Non-syndromic retinal ciliopathies				Syndromic ciliopathies with retinal degeneration														Other ciliopathies without retinal degeneration						
	**RP**	**CORD**	**LCA**	**MD**	**AS**	**BBS**	**CED**	**CILD**	**CRDHL**	**JBTS**	**MCCRP**	**MKS**	**MORM**	**MRCS**	**OFDS**	**SLS**	**SRTD**	**USH**	**COACH**	**LGMD**	**MKKS**	**NPHP**	**NSD**	**SGBS**	**References**
WDPCP						✓																			(Suspitsin and Imyanitov, 2016)

[a]From RetNet, OMIM, Syscilia, CiliaCarta and HGMD databases.

[b]AS, Alström syndrome; BBS, Bardet-Bield syndrome; CED, cranioectodermal dysplasia; CILD, ciliary dyskinesia; COACH, cerebellar vermis, hypo/aplasia, oligophrenia, congenital ataxia, ocular coloboma, and hepatic fibrosis; CORD, cone-rod dystrophy; CRDHL, cone-rod dystrophy and hearing loss; JBTS, Joubert syndrome; LCA, Leber congenital amaurosis; LGMD, limb-girdle muscular dystrophy; MCCRP, microcephaly and chorioretinopathy; MD, macular dystrophy; MKKS, McKusick-Kaufman syndrome; MKS, Meckel-Gruber syndrome; MORM, mental retardation, truncal obesity, retinal dystrophy, and micropenis; MRCS, microcornea-rod-cone dystrophy-cataract-posterior staphyloma syndrome; NPHP, nephronophthisis; NSD, non-syndromic deafness; OFDS, orofacialdigital syndrome; RP, retinitis pigmentosa; SGBS, Simpson-Golabi-Behmel syndrome; SLS, Senior-Løken syndrome; SRTD, short rib thoracic dysplasia with or without polydactyly; USH, Usher syndrome.

[c] Genes are listed in alphabetic order: non-syndromic retinal ciliopathies causative genes are in blue, genes whose mutations result in syndromic retinal ciliopathies are in orange, and genes which, depending on the mutation, can cause both non-syndromic and syndromic retinal ciliopathies are in dark red. Non-syndromic retinal ciliopathies are colored in light blue, syndromic ciliopathies with retinal degeneration are in light orange, and other ciliopathies that do not show retinal degeneration as a phenotype are in light purple.
